# Editorial: LuxR Solos are Becoming Major Players in Cell–Cell Communication in Bacteria

**DOI:** 10.3389/fcimb.2015.00089

**Published:** 2015-12-01

**Authors:** Vittorio Venturi, Brian M. M. Ahmer

**Affiliations:** ^1^International Centre for Genetic and BiotechnologyTrieste, Italy; ^2^Department of Microbial Infection and Immunity, The Ohio State UniversityColumbus, OH, USA; ^3^Center for Microbial Interface Biology, The Ohio State UniversityColumbus, OH, USA; ^4^Department of Microbiology, The Ohio State UniversityColumbus, OH, USA

**Keywords:** AHL, LuxR solos, quorum sensing, signaling, bacteria

Quorum sensing (QS) is the ability of microbes to sense and respond to their own population density, which typically results in cooperative activity (Fuqua et al., 1994). This form of microbial communication is important to agriculture and human health as they often participate in the regulation of genes important for host interactions.

The classic example of QS in bacteria is performed by the symbiotic bioluminescent bacterium *Vibrio fischeri* (Hastings and Greenberg, 1999). This bacterium colonizes the light organ of a squid and becomes luminescent at high population density. A pheromone of the acylhomoserine lactone class (AHL) is synthesized by the enzyme LuxI, and is a proxy for population density. The AHL is sensed by the transcription factor LuxR, which then activates the transcription of the *luxICDABEG* luciferase operon. Homologous LuxR-LuxI pairs have been found throughout the Proteobacteria (Fuqua et al., 2001); there is divergence among the structures of AHLs produced and detected by LuxI-LuxR pairs, providing some species specificity to the systems.

Several studies and the sequencing of many bacterial genomes has evidenced the presence of many AHL/QS-related *luxR*-type genes, which are unpaired to a cognate *luxI*. These LuxRs possess the typical modular structure having an AHL-binding domain and a DNA-binding HTH domain. These upaired *luxRs*/LuxRs have been called orphans (Fuqua, 2006; Patankar and Gonzalez, 2009) and more recently solos (Subramoni and Venturi, 2009). Several questions arise on the role of LuxR solos in bacteria and recent studies have revealed a number of roles including eavesdropping, intra-species and inter-kingdom signaling. This research topic of *Frontiers in Cellular and Infection Microbiology* is a collection of 10 articles which highlight these different roles as well as the widespread distribution of LuxR solos.

Three articles highlight how widespread LuxR solos are and provide data on their phylogenetic distribution (Gan et al., 2014; Hudaiberdiev et al., 2015; Subramoni et al., 2015). These surveys have shown the presence of one or multiple predicted LuxR solos in many proteobacterial genomes living in different environments, some of them also harboring genes for one or more complete AHL-QS circuits. LuxR solos can be tentatively clustered into meaningful groups or putative orthologs. These LuxR solos subfamilies could respond to different signals and/or having different roles.

The functions of solos can thus far be sub-divided in four categories; as detecting endogenous or exogenous signals, of either the classical AHL type, or of a novel type. The AHL-responsive solos can firstly detect exogenous AHLs, i.e., AHLs synthesized by other organisms, and this category is typified by the LuxR solo, SdiA (Sperandio, 2010; Soares and Ahmer, 2011; Swearingen et al., 2013; Sabag-Daigle et al., 2015). Orthologs of *sdiA* are present in *Escherichia, Salmonella, Enterobacter, Citrobacter, Cronobacter, Klebsiella, Pantoea*, and *Erwinia* (Sabag-Daigle and Ahmer, 2012). The *Pantoea* and *Erwinia* orthologs are part of *luxR-luxI* pairs and represent the ancestral state, and the *luxI* homolog was lost in the remaining genera, giving rise to the *sdiA* solos (Sabag-Daigle and Ahmer, 2012). In this issue, an *sdiA*-regulon study is presented showing a number of genes regulated by SdiA in *Enterobacter cloacae* (Sabag-Daigle et al., 2015). Interestingly some target genes were regulated in the complete absence of AHLs and thus AHLs may not be a “folding-switch” for SdiA, in which SdiA only folds correctly in the presence of AHL (Yao et al., 2006; Nguyen et al., 2015), AHL may alter the DNA binding specificity of SdiA so that there are AHL-dependent and AHL-independent promoters. LuxR solos can also be used to detect endogenous AHLs, i.e., AHLs that are made by the species detecting them. This “third wheel” type of LuxR solo is typified by QscR of *Pseudomonas aeruginosa* which harbors two complete AHL QS circuits, namely LasI/R and RhlI/R (Chugani and Greenberg, 2014; Martínez et al., 2015). In this issue, a review regarding the function of QscR is presented (Chugani and Greenberg, 2014; Martínez et al., 2015). QscR is involved in virulence and it responds to LasI generated AHLs, however it has a more relaxed specificity and is more promiscuous than LasR and its regulon overlaps with the one of LasR. The article particularly focuses on its biochemistry since QscR has become a model for understanding QS LuxR homologs.

LuxR solos can also respond to ligands which are not AHLs of either endogenous or exogenous sources. A large sub-family of LuxR solos has been found that specifically recognizes molecules from plants (González and Venturi, 2012; da Silva et al., 2015; Xu et al., 2015). These solos are only found in both pathogenic and beneficial plant-associated bacteria (PAB) and show changes in one or two highly conserved amino acids of the autoinducer binding domain (González and Venturi, 2012). Another member of this subfamily of PAB LuxR solos is reported in this *Frontiers* topic (Xu et al., 2015) as well as studies of protein domain switching between these solos and classic AHL responsive motifs (da Silva et al., 2015). A major step forward will be to identify the class of plant molecules that these solos respond to. Some solos respond to an endogenous, non-AHL, ligand. The LuxR-type receptor PluR of *Photorhabdus luminescens* responds to α-pyrones, while the related organism *Photorhabdus asymbiotica* responds to dialkylresorcinols using the LuxR homolog PauR (Brachmann et al., 2013; Brameyer et al., 2014, 2015; Brameyer and Heermann, 2015; Chen et al., 2015). The synthases for these molecules were determined to be PpyS and DarABC, respectively. In this instance, the LuxR solos turned out not to be solo at all. Instead, these LuxR homologs are paired with previously unrecognized types of synthases. In this topic, a survey of these LuxRs in *Photorhabdus* species is reported (Brameyer et al., 2014).

In summary, LuxR solos are widespread in Proteobacteria hence they are major players in bacterial communication and require more attention. Articles in this topic highlight the different modes of action of LuxR solos which are responding to endogenous and exogenous AHL or non-AHL signals. Further studies could lead to novel ways of controlling bacterial host colonization.

## Conflict of interest statement

The authors declare that the research was conducted in the absence of any commercial or financial relationships that could be construed as a potential conflict of interest.

## References

[B1] Brachmann A. O. Brameyer S. Kresovic D. Hitkova I. Kopp Y. Manske C.. (2013). Pyrones as bacterial signaling molecules. Nat. Chem. Biol. 9, 573–578. 10.1038/nchembio.1295 23851573

[B2] Brameyer S. Bode H. B. Heermann R. (2015). Languages and dialects: bacterial communication beyond homoserine lactones. Trends Microbiol. 23, 521–523. 10.1016/j.tim.2015.07.002 26231578

[B3] Brameyer S. Heermann R. (2015). Specificity of signal-binding via non-AHL LuxR-type receptors. PLoS ONE 10:e0124093. 10.1371/journal.pone.0124093 25923884 PMC4414361

[B4] Brameyer S. Kresovic D. Bode H. B. Heermann R. (2014). LuxR solos in Photorhabdus species. Front. Cell. Infect. Microbiol. 4:166. 10.3389/fcimb.2014.00166 25478328 PMC4235431

[B5] Chen R. Barphagha I. K. Ham J. H. (2015). Identification of potential genetic components involved in the deviant quorum-sensing signaling pathways of Burkholderia glumae through a functional genomics approach. Front. Cell. Infect. Microbiol. 5:22. 10.3389/fcimb.2015.00022 25806356 PMC4354385

[B6] Chugani S. Greenberg E. P. (2014). An evolving perspective on the *Pseudomonas aeruginosa* orphan quorum sensing regulator QscR. Front. Cell. Infect. Microbiol. 4:152. 10.3389/fcimb.2014.00152 25389523 PMC4211393

[B7] da Silva D. P. Patel H. K. González J. F. Devescovi G. Meng X. Covaceuszach S.. (2015). Studies on synthetic LuxR solo hybrids. Front. Cell. Infect. Microbiol. 5:52. 10.3389/fcimb.2015.00052 26151032 PMC4471428

[B8] Fuqua C. (2006). The QscR quorum-sensing regulon of *Pseudomonas aeruginosa*: an orphan claims its identity. J. Bacteriol. 188, 3169–3171. 10.1128/JB.188.9.3169-3171.2006 16621807 PMC1447470

[B9] Fuqua C. Parsek M. R. Greenberg E. P. (2001). Regulation of gene expression by cell-to-cell communication: acyl-homoserine lactone quorum sensing. Annu. Rev. Genet. 35, 439–468. 10.1146/annurev.genet.35.102401.090913 11700290

[B10] Fuqua W. C. Winans S. C. Greenberg E. P. (1994). Quorum sensing in bacteria: the LuxR-LuxI family of cell density-responsive transcriptional regulators. J. Bacteriol. 176, 269–275. 8288518 10.1128/jb.176.2.269-275.1994 PMC205046

[B11] Gan H. M. Gan H. Y. Ahmad N. H. Aziz N. A. Hudson A. O. Savka M. A. (2014). Whole genome sequencing and analysis reveal insights into the genetic structure, diversity and evolutionary relatedness of luxI and luxR homologs in bacteria belonging to the Sphingomonadaceae family. Front. Cell. Infect. Microbiol. 4:188. 10.3389/fcimb.2014.00188 25621282 PMC4288048

[B12] González J. F. Venturi V. (2012). A Novel Widespread Interkingdom Signaling Circuit. Trends Plant Sci. 18, 167–174. 10.1016/j.tplants.2012.09.007 23089307

[B13] Hastings J. W. Greenberg E. P. (1999). Quorum sensing: the explanation of a curious phenomenon reveals a common characteristic of bacteria. J. Bacteriol. 181, 2667–2668. 10217751 10.1128/jb.181.9.2667-2668.1999 PMC93702

[B14] Hudaiberdiev S. Choudhary K. S. Vera Alvarez R. Gelencsér Z. Ligeti B. Lamba D.. (2015). Census of solo LuxR genes in prokaryotic genomes. Front. Cell. Infect. Microbiol. 5:20. 10.3389/fcimb.2015.00020 25815274 PMC4357305

[B15] Martínez P. Huedo P. Martinez-Servat S. Planell R. Ferrer-Navarro M. Daura X.. (2015). *Stenotrophomonas maltophilia* responds to exogenous AHL signals through the LuxR solo SmoR (Smlt1839). Front. Cell. Infect. Microbiol. 5:41. 10.3389/fcimb.2015.00041 26029670 PMC4432800

[B16] Nguyen Y. Nguyen N. X. Rogers J. L. Liao J. MacMillan J. B. Jiang Y.. (2015). Structural and mechanistic roles of novel chemical ligands on the SdiA quorum-sensing transcription regulator. mBio 6:e02429-14. 10.1128/mBio.02429-14 25827420 PMC4453555

[B17] Patankar A. V. Gonzalez J. E. (2009). Orphan LuxR regulators of quorum sensing. FEMS Microbiol. Rev. 33, 739–756. 10.1111/j.1574-6976.2009.00163.x 19222586

[B18] Sabag-Daigle A. Ahmer B. M. (2012). ExpI and PhzI are descendants of the long lost cognate signal synthase for SdiA. PLoS ONE 7:e47720. 10.1371/journal.pone.0047720 23082201 PMC3474713

[B19] Sabag-Daigle A. Dyszel J. L. Gonzalez J. F. Ali M. M. Ahmer B. M. (2015). Identification of sdiA-regulated genes in a mouse commensal strain of *Enterobacter cloacae*. Front. Cell. Infect. Microbiol. 5:47. 10.3389/fcimb.2015.00047 26075189 PMC4444967

[B20] Soares J. A. Ahmer B. M. (2011). Detection of acyl-homoserine lactones by *Escherichia* and *Salmonella*. Curr. Opin. Microbiol. 14, 188–193. 10.1016/j.mib.2011.01.006 21353625 PMC3078957

[B21] Sperandio V. (2010). SdiA sensing of acyl-homoserine lactones by enterohemorrhagic *E. coli* (EHEC) serotype O157:H7 in the bovine rumen. Gut Microbes 1, 432–435. 10.4161/gmic.1.6.14177 21468228 PMC3056111

[B22] Subramoni S. Florez Salcedo D. V. Suarez-Moreno Z. R. (2015). A bioinformatic survey of distribution, conservation, and probable functions of LuxR solo regulators in bacteria. Front. Cell. Infect. Microbiol. 5:16. 10.3389/fcimb.2015.00016 25759807 PMC4338825

[B23] Subramoni S. Venturi V. (2009). LuxR-family ‘solos’: bachelor sensors/regulators of signalling molecules. Microbiology 155, 1377–1385. 10.1099/mic.0.026849-0 19383698

[B24] Swearingen M. C. Sabag-Daigle A. Ahmer B. M. (2013). Are there acyl-homoserine lactones within mammalian intestines? J. Bacteriol. 195, 173–179. 10.1128/JB.01341-12 23144246 PMC3553842

[B25] Xu H. Zhao Y. Qian G. Liu F. (2015). XocR, a LuxR solo required for virulence in *Xanthomonas oryzae* pv. oryzicola. Front. Cell. Infect. Microbiol. 5:37. 10.3389/fcimb.2015.00037 25932456 PMC4399327

[B26] Yao Y. Martinez-Yamout M. A. Dickerson T. J. Brogan A. P. Wright P. E. Dyson H. J. (2006). Structure of the *Escherichia coli* quorum sensing protein SdiA: activation of the folding switch by acyl homoserine lactones. J. Mol. Biol. 355, 262–273. 10.1016/j.jmb.2005.10.041 16307757

